# Six new species of *Horniella* Raffray from the Oriental region (Coleoptera, Staphylinidae, Pselaphinae)

**DOI:** 10.3897/zookeys.1042.66576

**Published:** 2021-06-04

**Authors:** Wen-Xuan Zhang, Fang-Shuo Hu, Zi-Wei Yin

**Affiliations:** 1 Laboratory of Systematic Entomology, College of Life Sciences, Shanghai Normal University, 100 Guilin Road, Xuhui District, Shanghai 200234, China Shanghai Normal University Shanghai China; 2 Department of Entomology, National Chung Hsing University 145 Xingda Rd., South District, Taichung City 402, Taiwan, China National Chung Hsing University Taichung China

**Keywords:** Ant-loving beetles, Asia, new record, new taxa, species list, taxonomy

## Abstract

The Oriental pselaphine genus *Horniella* Raffray, 1905 currently contains 29 species. In this paper, six new species are described: *H. nantouensis* Zhang, Hu & Yin, **sp. nov.** and *H. taiwanensis* Zhang, Hu & Yin, **sp. nov.** from Taiwan, China; *H. bifurca* Zhang & Yin, **sp. nov.** and *H. haucki* Zhang & Yin, **sp. nov.** from Thailand; *H. khasiensis* Zhang & Yin, **sp. nov.** from northern India; and *H. sabahensis* Zhang & Yin, **sp. nov.** from eastern Malaysia. In addition, *H. aculeata* Yin & Li, 2015, originally described from Yunnan, China, is newly recorded from Thailand.

## Introduction

The Oriental pselaphine genus *Horniella* Raffray, 1905 (Tyrini: Somatipionina) currently includes 29 species distributed in China (12 spp.), Thailand (9 spp.), Malaysia (4 spp.), Nepal and India (1 sp.), Sri Lanka (1 sp.), the Philippines (1 sp.), and Indonesia (1 sp.) (Raffray 1905; Yin and Li 2014, 2015; Newton 2020). Members of this genus are easily recognizable by their medium-sized to large body, enlarged maxillary palpomeres 4 that lack an apical palpal cone, presence of a frontal fovea on the head, weakly to greatly developed anterolateral genal projections, pronotum with median and lateral antebasal foveae that are connected by an antebasal sulcus, and usually medially carinate abdominal tergite 1 (IV) that is longer than tergite 2 (V). The known species were placed in four groups (Yin and Li 2014), which are followed here. The *H. centralis* group, with nine species, is defined by the distinct apicolateral genal projections, the head with a pair of long, curved ocular canthi, and the apical portion of the aedeagal median lobe with the right or left half strongly projecting apically. The *H. burckhardti* group, also containing nine species, is morphologically similar to the *H. centralis* group, but the apical portion of the aedeagal median lobe narrows apically. The *H. hirtella* group, represented by six species, lacks distinct apicolateral genal projections and ocular canthi, and the aedeagus usually has a relatively simple endophallus (membranous structures containing many small denticles). The *H. gigas* group, including three species, lacks obvious apicolateral genal projections or ocular canthi, each of the apical three antennomeres is distinctly elongate, maxillary palpomeres 2 are conspicuously elongate, tarsomeres 2 extend to near the midlength of tarsomeres 3, and the endophallus of the aedeagus has simple sclerites and/or small denticles on a membranous structure. A large number of unassociated females have been listed by Yin and Li (2014, 2015), which indicates that the true diversity of this group still remains underexplored.

Based on an examination of additional material deposited in the Muséum d’Histoire Naturelle, Geneva, Switzerland, and the National Museum of Natural Science, Taichung City, Taiwan, China, we describe here six new species from China (2), Thailand (2), India (1), and Malaysia (1). Thus, the total species number of *Horniella* raises from 29 to 35. Furthermore, new collecting data of *Horniella aculeata* Yin & Li from Thailand are provided.

## Material and methods

The type material of the new species described in this paper is deposited in the Muséum d’Histoire Naturelle, Geneva (**MHNG**), the National Museum of Natural Science, Taichung City, TaiwanTaiwan (**NMNS**), and the Insect Collection of Shanghai Normal University, Shanghai (**SNUC**).

Dissected parts were preserved in Euparal on plastic slides that were placed on the same pin with the specimen. The habitus images of the beetles were taken using a Canon 5D Mark III camera in conjunction with a Canon MP-E 65 mm f/2.8 1–5× macro lens, and a Canon MT-24EX Macro Twin Lite flash was used as the light source. Images of the morphological details were produced using a Canon G9 camera mounted to an Olympus CX31 microscope under reflected or transmitted light. Zerene Stacker v. 1.04 was used for image stacking. All images were modified and grouped into plates using Adobe Photoshop CC 2020.

The abdominal tergites and sternites are numbered following Chandler (2001) in Arabic (starting from the first visible segment) and Roman (reflecting true morphological position) numerals, e.g., tergite 1 (IV), or sternite 1 (III). Paired structures in the species descriptions are treated as singular.

The collecting data of the material are quoted verbatim. The Chinese translation of each locality is included in parentheses at first appearance in the text. A slash is used to separate different labels. Each type specimen bears the following label: ‘HOLOTYPE [red] (or PARATYPE [yellow]), ♂ (or ♀), *Horniella* + specific name sp. n., det. Zhang & Yin, 2021, NMNS (or MHNG, or SNUC)’.

The following abbreviations are applied: **AL** = length of the dorsally exposed part of the abdomen (posterior to elytra) along the midline; **AW** = maximum width of the abdomen; **EL** = length of the elytra along the suture; **EW** = maximum width of the elytra; **HL** = length of the head from the anterior clypeal margin to the anterior margin of the occipital constriction; **HW** = width of the head across eyes; **PL** = length of the pronotum along the midline; **PW** = maximum width of the pronotum. Length of the body is a sum of HL + PL + EL + AL.

## Results

### Horniella aculeata

Taxon classificationAnimaliaAmphipodaHornelliidae

Yin & Li, 2015

61B0B45F-6D2C-55F0-A5AC-A2ECF7782C1E

Horniella aculeata Yin & Li, 2015: 110.

#### Material examined.

2 ♂♂, labeled ‘Thailand: Nan prov. Doi Phuka Nat. Park, 28.IV-12.V.2002, Průdek & Obořil lgt.’ (MHNG); 1 ♂, labeled ‘THAI, 28–31.v.1995, 19.27N, 98.20E, SOPPONG 1500 m, Vit Kubáň leg.’ (MHNG).

#### Remarks.

*Horniella aculeata* is readily recognizable by the presence of a large spine on the mesal margin of the protibiae (Yin and Li 2015: fig. 2F), and the aedeagus with one elongate, twisted sclerite (Yin and Li 2015: fig. 2K). This species was described based on two male and four female specimens from Yunnan, China, and the present record extends its distribution to Thailand.

#### Distribution.

China: Yunnan; Thailand: Nan, Mae Hong Son. **New country record for Thailand.**

### Horniella bifurca

Taxon classificationAnimaliaAmphipodaHornelliidae

Zhang & Yin sp. nov.

948F301A-56FB-5616-BAF2-61004D6D59D2

http://zoobank.org/DD97E152-F782-4FC5-8B59-27D1D383C7A9

**Figure 1.**
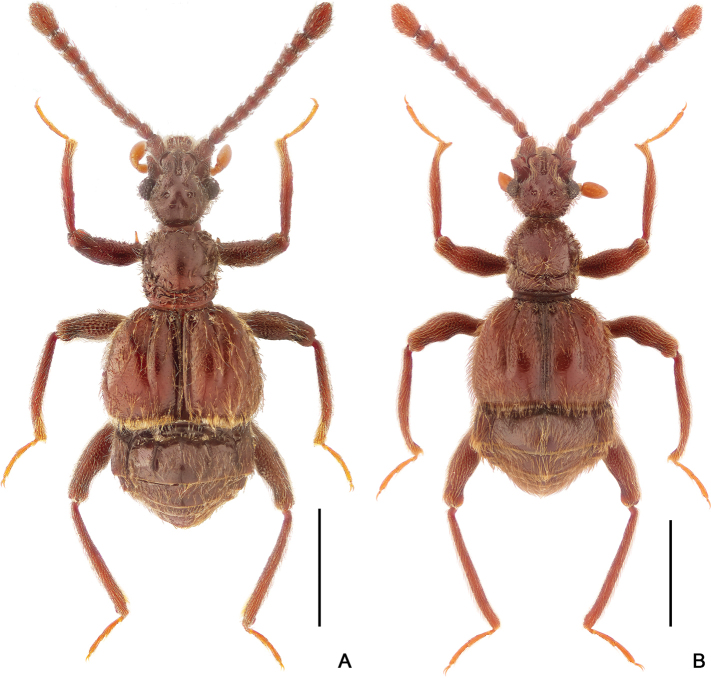
Dorsal habitus of *Horniella* species **A***H. bifurca* sp. nov. **B***H. haucki* sp. nov. Scale bars: 1 mm.

**Figure 2.**
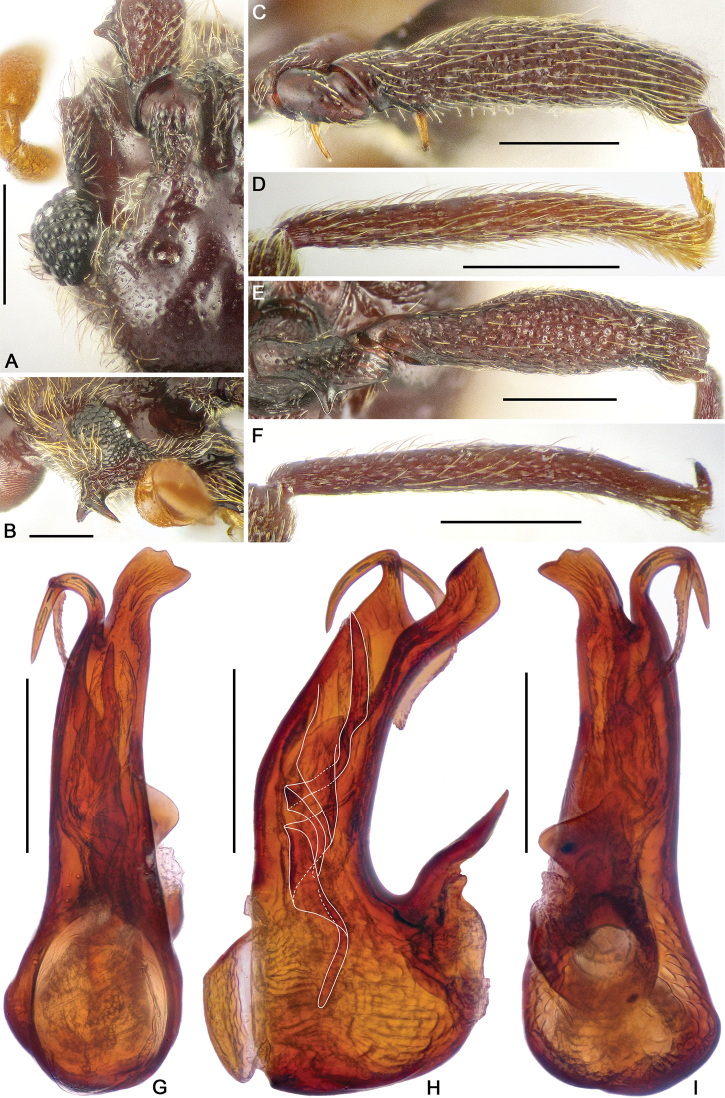
Diagnostic characters of *Horniella bifurca* sp. nov. **A** left half of head, in dorsal view **B** head, in lateral view **C** protrochanter and profemur **D** protibia **E** mesotrochanter and mesofemur **F** mesotibia **G–I** aedeagus, in dorsal (**G**), lateral (**H**), and ventral (**I**) view. Scale bars: 0.3 mm (**A, C–F**); 0.2 mm (**B, G–I**).

Figures 1A , 2 , 10A

#### Type material.

***Holotype*: Thailand**: ♂, ‘THAILAND: Chiang Mai, Pinh Khong env. 900 m, 19°26.70'N, 99°01.9'E, 14.xi.2012, M. Košťál lgt.’ (MHNG). ***Paratype*: Thailand**: 1 ♀, same label data as for holotype (MHNG).

#### Diagnosis.

**Male.** Head approximately as long as wide, with distinct anterolateral genal projection, anterior margin of projection roundly emarginate; with long, apically forked ocular canthus; scape angularly expanded at anterolateral margin, antennomeres 9–11 moderately enlarged. Pronotum rounded at anterolateral margins. Protrochanter and profemur each with long ventral spine; protibia with small triangular apical spur; mesotrochanter with short but distinct ventral spine. Tergite 1 (IV) with median carina extending posteriorly for approximately 1/4 of tergal length, discal carinae long and thick. Aedeagus with asymmetric median lobe, right half of median lobe greatly protruding apicad, left half strongly curved and forked at apex; endophallus composed of two elongate, twisted sclerites.

**Female.** Similar to male in external morphology, profemur each with two ventral spines near base, protibia lacking preapical spur, mesotrochanter lacking ventral spine; genital complex as in Fig. 10A.

#### Description.

**Male.** Body reddish-brown, length 3.35 mm. Head (Fig. 2A) approximately as long as wide, HL 0.69 mm, HW 0.7 mm; anterolateral genal projection distinct, anterior margin of projection roundly emarginate; antenna 1.7 mm long, scape angularly expanded at anterolateral margin, antennomeres 2–8 slightly elongate or moniliform, club loosely formed by apical three moderately enlarged antennomeres, antennomere 11 largest, approximately as long as antennomeres 9 and 10 combined; indistinct lateral postantennal pits present; eyes prominent, each composed of approximately 45 large facets, with long, broad forked ocular canthi (Fig. 2B).

Pronotum as long as wide, PL and PW 0.67 mm, widest at apical 1/3; anterolateral margins rounded; disc moderately convex, finely punctate, with distinct median antebasal and lateral antebasal foveae connected by complete transverse sulcus.

Elytra much wider than long, EL 0.91 mm, EW 1.36 mm; each elytron with two large, setose basal foveae; discal striae extending from outer basal foveae to apical 3/4 of elytral length.

Legs elongate; protrochanter (Fig. 2C) with elongate ventral spine, profemur (Fig. 2C) with long ventral spine near base, protibia (Fig. 2D) with small triangular apical spur; mesotrochanter (Fig. 2E) with short but distinct ventral spine, mesofemur (Fig. 2E) and mesotibia (Fig. 2F) simple.

Abdomen slightly broader than long, broadest at lateral margins of tergite 1 (IV), AL 1.08 mm, AW 1.22 mm; tergite 1 (IV) largest, as long as tergites 2 and 3 (V and VI) combined, with short median carina extending to near basal 1/4 of tergal length, discal carinae long and thick, with broad basal impression, tergite 2 (V) lacking carina, tergites 2–4 (V–VII) each with small basolateral foveae. Sternite 2 (IV) with broad basal sulcus, lacking mediobasal foveae, basolateral foveae developed as large cuticular pockets, with two pairs of antebasal protuberances, sternites 3–5 (V–VII) each with basolateral foveae, and one median and two lateral nodules, sternite 7 (IX) with well-sclerotized apical half and membranous basal half.

Aedeagus (Fig. 2G–I) 0.61 mm long, with asymmetric median lobe, right half of median lobe greatly protruding apicad, left half elongate, with strongly curved and deeply forked apical part; endophallus composed of two elongate, twisted sclerites.

**Female.** General morphology similar to male, each eye composed of approximately 30 facets; profemur with two long ventral spines near base, protibia lacking preapical spur, mesotrochanter lacking ventral spine. Measurements (as for male): BL 3.08 mm, HL 0.68 mm, HW 0.61 mm, PL 0.65 mm, PW 0.63 mm, EL 0.79 mm, EW 1.17 mm, AL 0.96 mm, AW 1.2 mm. Genital complex (Fig. 10A) with transverse apical sclerite, and elongate membranous basal portion.

#### Comparative notes.

This species is placed as a member of the *H. centralis* group. It can be readily separated from the other members of the group by the long, apically-forked ocular canthi, as well as by the unique shape of the aedeagus.

#### Distribution.

Thailand: Chiang Mai.

#### Etymology.

The new specific epithet *bifurca* (-*us*, -*um*) is a Latin adjective means ‘two-pronged’, referring to the apically-forked ocular canthus of the new species.

### Horniella haucki

Taxon classificationAnimaliaAmphipodaHornelliidae

Zhang & Yin sp. nov.

F95EBCEF-255E-52F3-9143-6BF7DD3C8B6A

http://zoobank.org/4A4C1B4E-F834-4742-BD77-CA7879983132

Figures 1B , 3

#### Type material.

***Holotype*: Thailand**: ♂, ‘THAI, N, Mae Hong Son prov., SE of Soppong, 1500 m, 19°27'N, 98°20'E, 23–27.v.1999, D. Hauck leg.’ (MHNG).

#### Diagnosis.

**Male.** Head longer than wide, with distinct anterolateral genal projection, anterior margin of projection roundly emarginate; with long ocular canthus; scape angularly expanded at basolateral margin, antennomeres 9–11 moderately enlarged. Pronotum rounded at anterolateral margins. Protrochanter and profemur each with long ventral spine; protibia strongly curved near apex, with long apical projection; mesotrochanter with large sharp ventral spine, mesofemur distinctly arched. Tergite 1 (IV) with median carina extending posteriorly for approximately 3/4 of tergal length, discal carinae short and thin. Aedeagus with asymmetric median lobe, left half of median lobe greatly protruding in dorso-ventral view; endophallus composed of three long sclerites.

#### Description.

**Figure 3.**
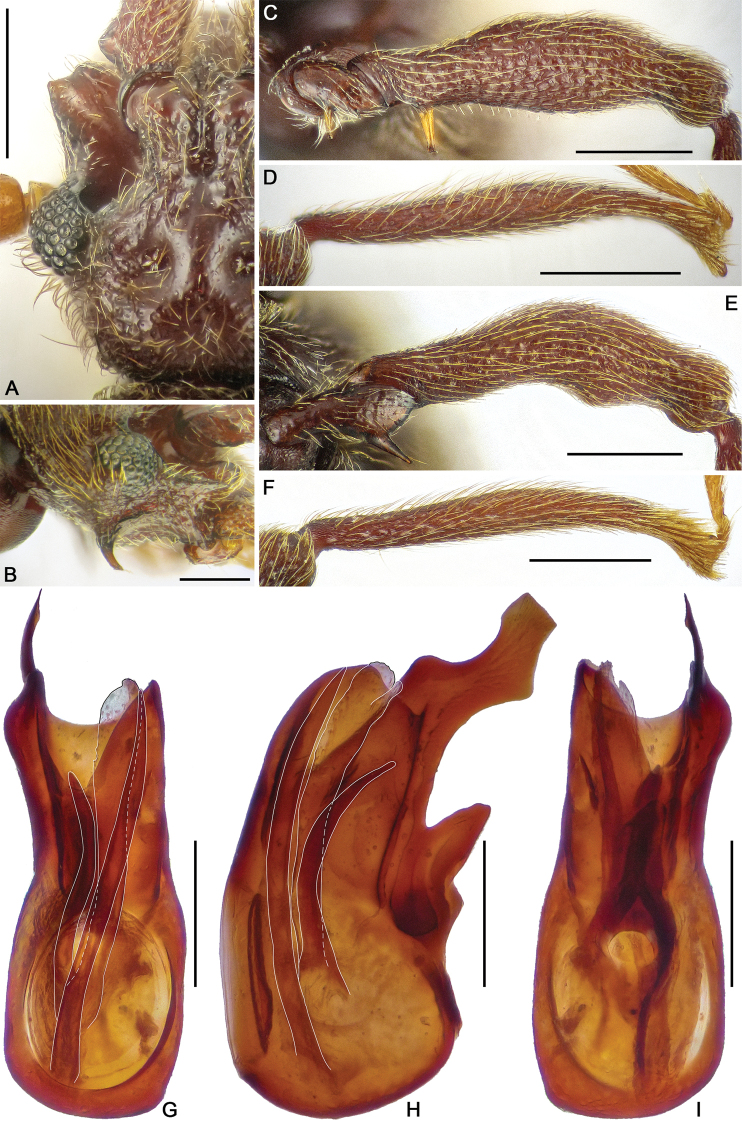
Diagnostic characters of *Horniella haucki* sp. nov. **A** left half of head, in dorsal view **B** head, in lateral view **C** protrochanter and profemur **D** protibia **E** mesotrochanter and mesofemur **F** mesotibia **G–I** aedeagus, in dorsal (**G**), lateral (**H**), and ventral (**I**) view. Scale bars: 0.3 mm (**A, C–F**); 0.2 mm (**B, G–I**).

**Male.** Body reddish-brown, length 3.49 mm. Head (Fig. 3A) slightly longer than wide, HL 0.75 mm, HW 0.7 mm; anterolateral genal projection distinct, anterior margin of projection roundly emarginate; antenna 1.95 mm long, scape angularly expanded at basolateral margin, antennomeres 2–7 slightly elongate, antennomere 8 as long as wide, club loosely formed by apical three moderately enlarged antennomeres, antennomere 11 largest, approximately as long as antennomeres 9 and 10 combined; indistinct lateral postantennal pits present; eyes prominent, each composed of approximately 40 large facets, with long ocular canthi (Fig. 3B).

Pronotum longer than wide, PL 0.74 mm, PW 0.69 mm, widest at apical 1/3; anterolateral margins rounded; disc moderately convex, finely punctate, with distinct median antebasal and lateral antebasal foveae connected by complete transverse antebasal sulcus.

Elytra much wider than long, EL 0.95 mm, EW 1.35 mm; each elytron with two large, setose basal foveae; discal striae extending from outer basal foveae to apical 2/3 of elytral length.

Legs elongate; protrochanter (Fig. 3C) with distinct ventral spine, profemur (Fig. 3C) with long ventral spine near base, protibia (Fig. 3D) strongly curved near apex, with long apical projection; mesotrochanter (Fig. 3E) with long sharp ventral spine, mesofemur (Fig. 3E) strongly arched at middle, mesotibia (Fig. 3F) strongly curved near apex, with small triangular spur.

Abdomen slightly broader than long, broadest at lateral margins of tergite 1 (IV), AL 1.05 mm, AW 1.32 mm; tergite 1 (IV) largest, as long as tergites 2 and 3 (V and VI) combined, with median carina extending to near basal 3/5 of tergal length, discal carinae short and thin, tergite 2 (V) lacking carina, tergites 2–4 (V–VII) each with small basolateral foveae. Sternite 2 (IV) with broad basal sulcus, lacking mediobasal foveae, basolateral foveae developed as large cuticular pockets, with two pairs of antebasal nodules, sternites 3–5 (V–VII) with basolateral foveae, one median and two lateral nodules, sternite 7 (IX) with well-sclerotized apical half, and membranous basal half.

Aedeagus (Fig. 3G–I) 0.69 mm long, with asymmetric median lobe, left half of median lobe greatly protruding in dorsal view; endophallus composed of three elongate sclerites close to each other.

**Female.** Unknown.

#### Comparative notes.

This new species can be readily separated from all members of the *H. centralis* group primarily by the characteristic shape of the aedeagus, especially the form of the apical portion of the median lobe, and the configuration of the endophallus.

#### Distribution.

Thailand: Mae Hong Son.

#### Etymology.

The new species is named after David Hauck (České Budějovice, Czech Republic), collector of the holotype.

### Horniella khasiensis

Taxon classificationAnimaliaAmphipodaHornelliidae

Zhang & Yin sp. nov.

60E9CC75-AF9D-58E8-8E3C-DC2CE141887F

http://zoobank.org/7DC18538-F16A-4665-ADAA-342B93D6D713

Figures 4A , 5

#### Type material.

***Holotype*: India**: ♂, ‘INDIA, Meghalaya State (7+9), E Khasi Hills, 11km SW Cherrapunjee, Laitkynsew, 25.iv.2008, 25°12'48"N, 91°39'48"E, 735 m, Fikáček, Podskalská, Šípek lgt. / secondary tropical rainforest with young trees + bamboo, below village, thin layer of leaf litter (sifting).’ (MHNG).

**Figure 4.**
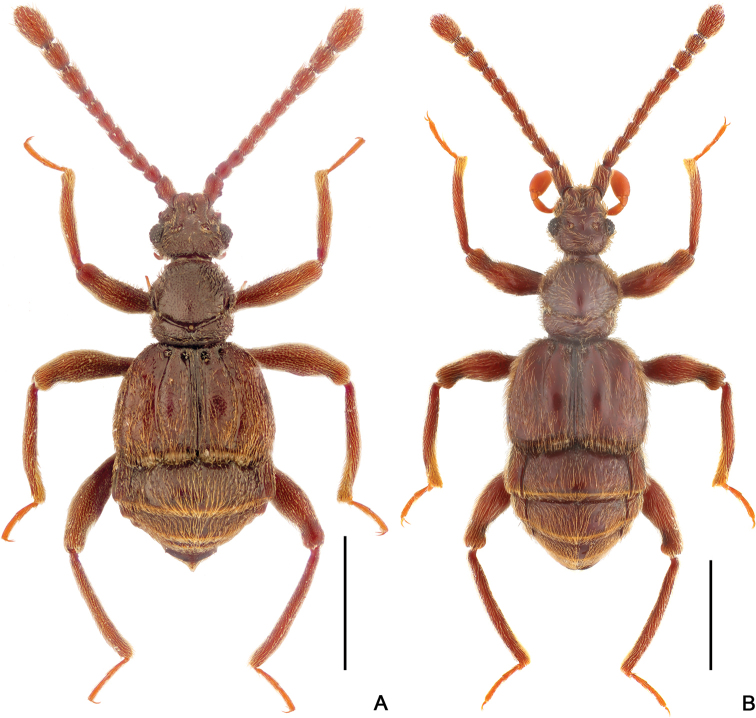
Dorsal habitus of *Horniella* species **A***H. khasiensis* sp. nov. **B***H. nantouensis* sp. nov. Scale bars: 1 mm.

#### Diagnosis.

**Male.** Head wider than long, with distinct anterolateral genal projection, anterior margin of projection roundly emarginate; with markedly long ocular canthus; scape angularly expanded at middle of lateral margin, antennomeres 9–11 enlarged. Pronotum rounded at anterolateral margins. Protrochanter and profemur each with long ventral spine; mesotrochanter with short, small ventral tubercle. Tergite 1 (IV) with median carina extending posteriorly for approximately 1/4 of tergal length, lacking discal carinae, tergite VIII with large medioapical process. Aedeagus with slightly asymmetric median lobe, apex broadly truncate in dorso-ventral view; endophallus composed of three sclerites.

#### Description.

**Figure 5.**
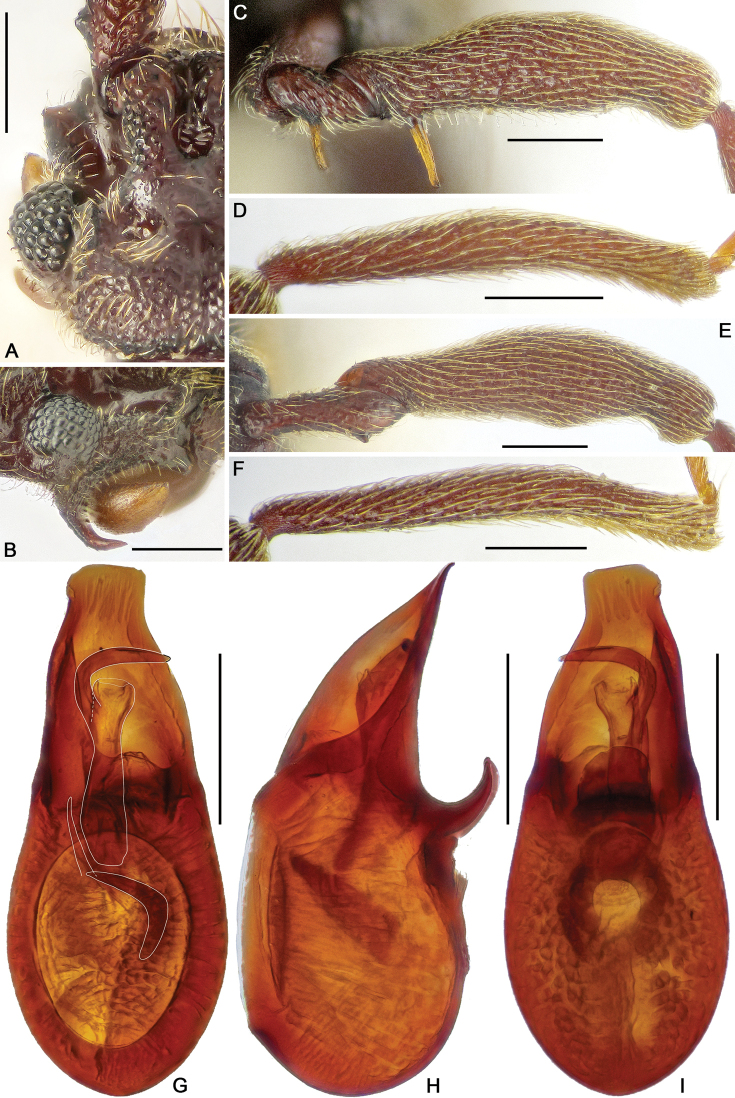
Diagnostic characters of *Horniella khasiensis* sp. nov. **A** left half of head, in dorsal view **B** head, in lateral view **C** protrochanter and profemur **D** protibia **E** mesotrochanter and mesofemur **F** mesotibia **G–I** aedeagus, in dorsal (**G**), lateral (**H**) and ventral (**I**) view. Scale bars: 0.2 mm (**A–I**).

**Male.** Body reddish-brown, length 2.84 mm. Head (Fig. 5A) wider than long, HL 0.56 mm, HW 0.63 mm; anterolateral genal projection distinct, anterior margin of projection roundly emarginate; antenna 1.85 mm long, scape angularly expanded at middle of lateral margin, antennomeres 2–8 slightly elongate or moniliform, club loosely formed by apical three moderately enlarged antennomeres, antennomere 11 largest, slightly shorter than antennomeres 9 and 10 combined; indistinct lateral postantennal pits present; eyes prominent, each composed of approximately 40 large facets, with markedly long and curved ocular canthi (Fig. 5B).

Pronotum as long as wide, PL and PW 0.64 mm, widest anterior to middle; lateral margins rounded; disc moderately convex, finely punctate, with distinct median antebasal and lateral antebasal foveae connected by complete transverse antebasal sulcus.

Elytra much wider than long, EL 0.75 mm, EW 1.2 mm; each elytron with two large, setose basal foveae; discal striae extending from outer basal foveae to near posterior margin of elytra.

Legs elongate; protrochanter (Fig. 5C) with elongate ventral spine, profemur (Fig. 5C) with conspicuously long ventral spine near base, protibia (Fig. 5D) simple; mesotrochanter (Fig. 5E) with short and small ventral tubercle, mesofemur (Fig. 5E) and mesotibia (Fig. 5F) simple.

Abdomen broader than long, broadest at lateral margins of tergite 1 (IV), AL 0.89 mm, AW 1.21 mm; tergite 1 (IV) largest, as long as tergites 2 and 3 (V and VI) combined, with short median carina extending to near basal 1/4 of tergal length, lacking discal carinae, tergite 2 (V) lacking carina, tergites 2–4 (V–VII) each with small basolateral foveae, tergite 5 (VIII) with large medioapical process. Sternite 2 (IV) with broad basal sulcus, lacking mediobasal foveae, basolateral foveae developed as large cuticular pockets, with two pairs of antebasal nodules, sternites 3–5 (V–VII) with basolateral foveae, one median and two lateral nodules, sternite 7 (IX) nearly oval, with well-sclerotized apical half and less sclerotized basal half.

Aedeagus (Fig. 5G–I) 0.59 mm long, median lobe nearly symmetric, apex broadly truncate; endophallus composed of three sclerites: one elongate, plate-like sclerite with curved lobe at apex; one curved sclerite at base, and one much narrower sclerite at left.

**Female.** Unknown.

#### Comparative notes.

This species is placed as a member of the *H. burckhardti* group, and is most similar to *H. hongkongensis* Yin & Li in having similar spination of the legs and a general aedeagal form. They can be clearly separated by the more distinctly expanded basolateral margin of the scape, tergite VIII with a large medioapical process, and the different structure of the aedeagal endophallus.

#### Distribution.

India: Meghalaya.

#### Etymology.

The new species is named after its type locality, the East Khasi Hills.

### Horniella nantouensis

Taxon classificationAnimaliaAmphipodaHornelliidae

Zhang, Hu & Yin sp. nov.

230218FD-1216-5309-9DAC-3E7E6D46147E

http://zoobank.org/BAC47C36-A9D6-4D70-9A6D-2B5281C4904F

**Figure 6.**
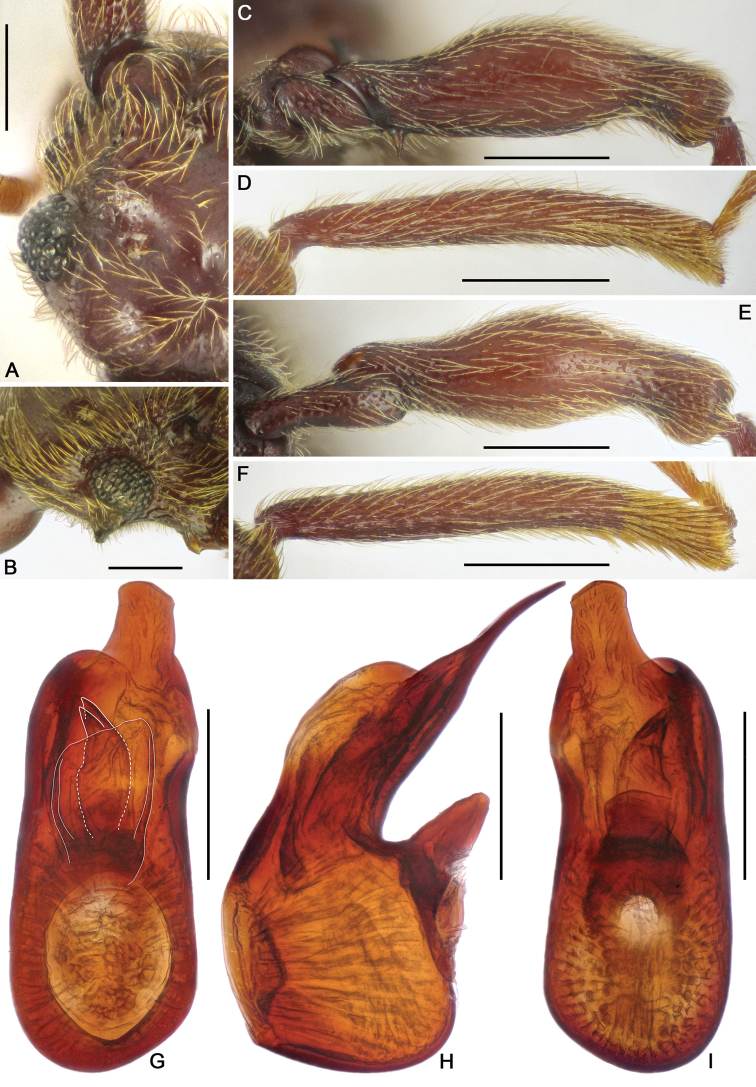
Diagnostic characters of *Horniella nantouensis* sp. nov. **A** left half of head, in dorsal view **B** head, in lateral view **C** protrochanter and profemur **D** protibia **E** mesotrochanter and mesofemur **F** mesotibia **G–I** aedeagus, in dorsal (**G**), lateral (**H**), and ventral (**I**) view. Scale bars: 0.2 mm (**A, B, G–I**); 0.3 mm in (**C–F**).

Figures 4B , 6 , 10B

#### Type material.

***Holotype*: China**: ♂, ‘TAIWAN: Nantou County, Huisun Forest Reserve [惠荪林场], track to Xiaochushan Mt., 24.0745N, 121.0366E; 1150 m, 4.v.2019; Damaška, Fikáček, Hu & Liu lgt., 2019-TW14 / primary forest on the slope with sparse understory; sifting of small accumulations of leaves / Huisun Leaf Litter Beetles Project, Additional specimen: HS1-034 / *HORNIELLA* sp., P. Hlaváč det., 2019’ (NMNS). ***Paratypes*: China**: 1 ♀, same data as holotype, except ‘Huisun Leaf Litter Beetles Project, Additional specimen: HS1-035’ (NMNS); 1 ♀, ‘same locality and date, except ‘24.0826N, 121.0316E; 1050 m, 2019-TW15’ / sparse secondary forest with dense understory incl. tree ferns on the margin of a tree plantation: sifting / Huisun Leaf Litter Beetles Project, Additional specimen: HS2-041’ (NMNS); 1 ♂, ‘Tehuashe (900 m), Nantou, Taiwan, 南投县德化社 (Tehuashe), 14.xi.2000, Hiroshi Sugaya leg. (in the leaf litter)’ (MHNG); 1 ♀, same data as previous, except ‘4–5.v.(20)01’ (MHNG); 2 ♀♀, ‘Aowanta (1400 m), Nantou, Taiwan, 南投县奥万大, 15.xi.2000, Hiroshi Sugaya leg. (in the leaf litter)’ (MHNG).

#### Diagnosis.

**Male.** Head longer than wide, with weakly indicated anterolateral genal projection, anterior margin of projection oblique; with short ocular canthus; lateral margin of scape straight, antennomeres 9–11 slightly enlarged. Pronotum rounded at anterolateral margins. Ventral margin of profemur with one short and acute, and one tiny spine at base; protibia with one small preapical denticle. Tergite 1 (IV) with median carina extending posteriorly for approximately 1/3 of tergal length, lacking discal carinae. Aedeagus with asymmetric median lobe, apical part of median lobe narrowed and protruding apicad, apex nearly rounded in dorsal view. Female. Similar to male in external morphology, profemur with two ventral spines near base; genital complex as in Fig. 10B.

#### Description.

**Male.** Body reddish-brown, length 3.68 mm. Head (Fig. 6A) slightly longer than wide, HL 0.74 mm, HW 0.63 mm; anterolateral genal projection weakly developed, anterior margin of projection oblique; antenna 2.03 mm long, scape lacking expansion at lateral margin, antennomeres 2–8 slightly elongate or moniliform, club loosely formed by apical three moderately enlarged antennomeres, antennomere 11 largest, as long as antennomeres 9 and 10 combined; indistinct lateral postantennal pits present; eyes prominent, each composed of approximately 45 large facets, with pair of short ocular canthi (Fig. 6B).

Pronotum longer than wide, PL 0.77 mm, PW 0.71 mm; widest at apical 1/3; anterolateral margins rounded; disc moderately convex, finely punctate, with distinct median antebasal and lateral antebasal foveae connected by complete transverse antebasal sulcus.

Elytra much wider than long, EL 0.85 mm, EW 1.33 mm; each elytron with two large, setose basal foveae; discal striae extending from outer basal foveae to middle of elytral length.

Legs elongate; protrochanter (Fig. 6C) simple, profemur (Fig. 6C) with one short and acute, and one tiny ventral spine at base; protibia (Fig. 6D) with small preapical spur; mesotrochanter (Fig. 6E) and mesofemur (Fig. 6E) simple, mesotibia (Fig. 6F) with thick apical setae.

Abdomen slightly longer than broad, broadest at lateral margins of tergite 1 (IV), AL 1.32 mm, AW 1.28 mm; tergite 1 (IV) slightly longer than tergites 2 (V), with median carina extending to near basal 1/3 of tergal length, lacking discal carinae, tergite 2 (V) lacking carina, tergites 2–4 (V–VII) each with small basolateral foveae. Sternite 2 (IV) with broad basal sulcus, lacking mediobasal foveae, basolateral foveae developed as large cuticular pockets, with two pairs of antebasal nodules, sternites 3–5 (V–VII) with basolateral foveae, one median and two lateral nodules.

Aedeagus (Fig. 6G–I) 0.57 mm long, with asymmetric median lobe, apical portion of median lobe narrowed and greatly protruding apically, apex nearly rounded in dorsal view; endophallus composed of broad membranous part with single broad elongate sclerite at middle.

**Female.** General morphology similar to male, each eye composed of approximately 40 facets; profemur each with two distinct ventral spines near base, protibia lacking spur. Measurements (as for male): BL 3.68–3.72 mm, HL 0.74 mm, HW 0.63–0.65 mm, PL 0.72–0.73 mm, PW 0.74–0.75 mm, EL 0.87–0.9 mm, EW 1.33 mm, AL 1.35 mm, AW 1.32–1.33 mm. Genital complex (Fig. 10B) with moderately sclerotized central and membranous lateral parts.

#### Comparative notes.

This species is placed as a member of the *H. hirtella* group. The new species is similar to *H. simplaria* Yin & Li by the male having similar anterolateral genal projections, and presence of two ventral spines of profemur. They can be otherwise clearly separated by the larger body size (3.68 mm vs 3.23 mm), lack of a mesal hook-like spine of the protibia (present in *H. simplaria*), and the different shape and structure of the aedeagus of the new species.

#### Distribution.

China: Taiwan.

#### Etymology.

The new specific is named after its type locality, Nantou County.

### Horniella sabahensis

Taxon classificationAnimaliaAmphipodaHornelliidae

Zhang & Yin sp. nov.

BC7F7BA6-A026-57A0-AB24-449D96A75047

http://zoobank.org/EC5C6E94-0D2A-461D-8EA7-1C750F093066

Figures 7A , 8

#### Type material.

***Holotype*: East Malaysia**: ♂, ‘Borneo: Sabah, Batu Punggul Resort, primary forest, 24.vi.–1.vii.96, Kodada lgt. / vegetation debris and forest floor litter accumulated around large trees near river.’ (MHNG).

**Figure 7.**
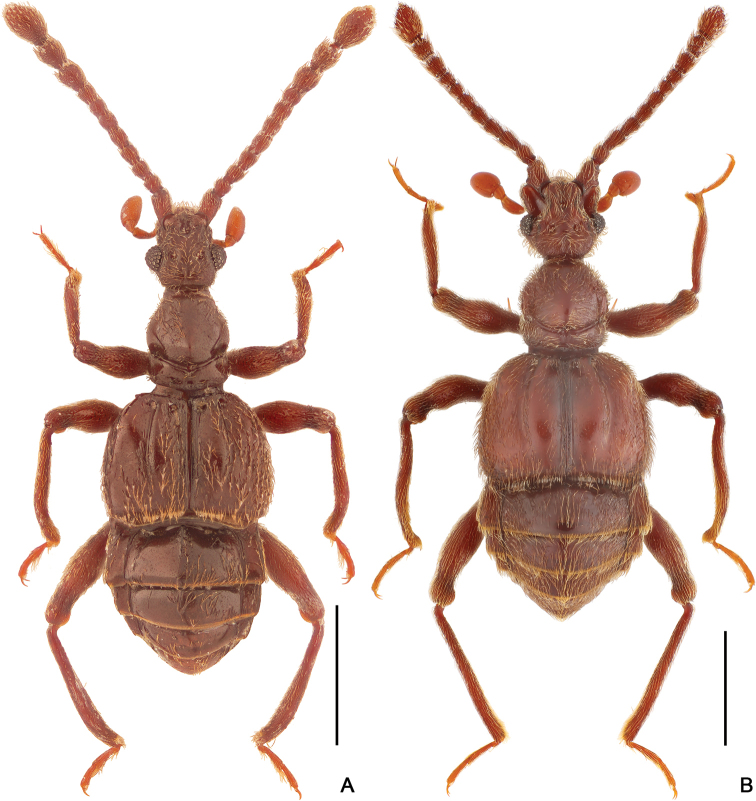
Dorsal habitus of *Horniella* species **A***H. sabahensis* sp. nov. **B***H. taiwanensis* sp. nov. Scale bars: 1 mm.

#### Diagnosis.

**Male.** Head longer than wide, anterolateral genal projections weakly developed, anterior margin of projection oblique; scape lacking expansion at lateral margin, antennomeres 9–11 moderately enlarged, forming distinct club. Pronotum rounded at lateral margins. Profemur with two tiny ventral spines near base; metatibia with preapical triangular denticle. Tergite 1 (IV) with median carina extending posteriorly for approximately 3/4 of tergal length, lacking discal carinae, tergite 2 (V) with short median carina. Aedeagus with slightly asymmetric median lobe, apical portion of median lobe narrowed, apex truncate in dorso-ventral view; endophallus lacking sclerite, composed of elongate membranous structure with many small denticles.

#### Description.

**Figure 8.**
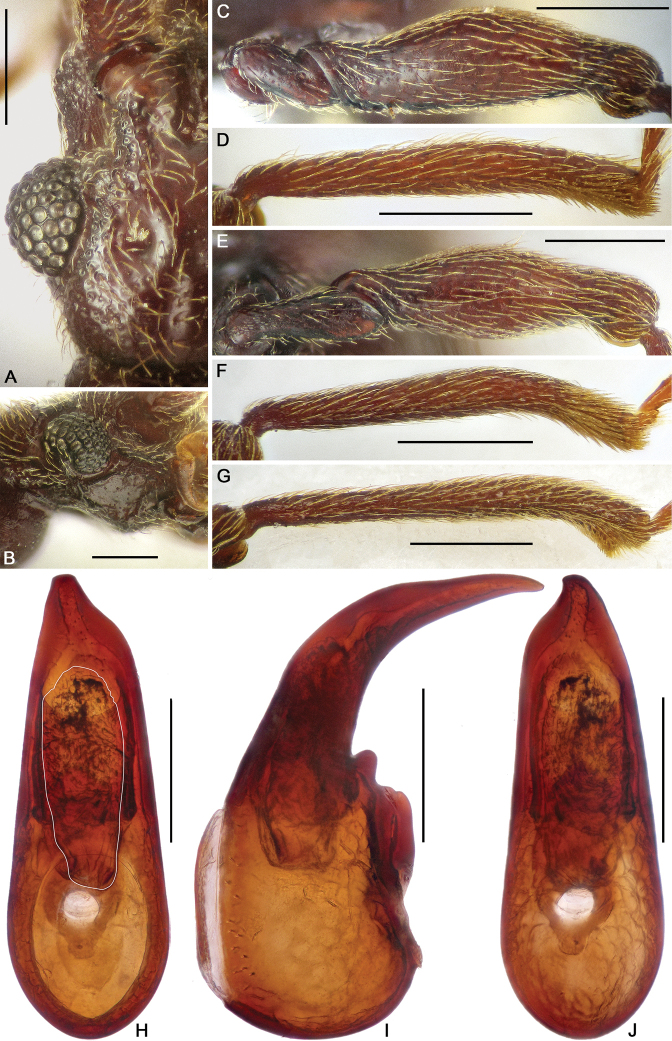
Diagnostic characters of *Horniella sabahensis* sp. nov. **A** left half of head, in dorsal view **B** head, in lateral view **C** protrochanter and profemur **D** protibia **E** mesotrochanter and mesofemur **F** mesotibia **G** metatibia **H–J** aedeagus, in dorsal (**H**), lateral (**I**), and ventral (**J**) view. Scale bars: 0.2 mm (**A, B, H–J**); 0.3 mm (**C–G**).

**Male.** Body reddish-brown, length 3.41 mm. Head (Fig. 8A) longer than wide, HL 0.68 mm, HW 0.59 mm; anterolateral genal projection weakly developed, anterior margin of projection oblique; antenna 1.96 mm long, scape lacking expansion at lateral margin, antennomeres 2–8 slightly elongate or moniliform, distinct club formed by apical three enlarged antennomeres, antennomere 11 largest, slightly shorter than antennomeres 9 and 10 combined; indistinct lateral postantennal pits present; eyes prominent, each composed of approximately 40 large facets, usual area of ocular canthus only slightly prominent (Fig. 8B).

Pronotum distinctly longer than wide, PL 0.71 mm, PW 0.6 mm; widest at middle; lateral margins rounded; disc moderately convex, finely punctate, with distinct median antebasal and lateral antebasal foveae connected by complete transverse antebasal sulcus.

Elytra much wider than long, EL 0.77 mm, EW 1.2 mm; each elytron with two large, setose basal foveae; discal striae extending from outer basal foveae to apical 2/3 of elytral length.

Legs elongate; protrochanter (Fig. 8C) simple, profemur (Fig. 8C) with two tiny ventral spines at base, protibia (Fig. 8D) simple; mesotrochanter, mesofemur (Fig. 8E) and mesotibia (Fig. 8F) simple; metatibia (Fig. 8G) with distinct apical triangular denticle.

Abdomen slightly longer than broad, broadest at lateral margins of tergite 1 (IV), AL 1.25 mm, AW 1.16 mm; tergite 1 (IV) largest, slightly shorter than tergites 2 and 3 (V and VI) combined, with median carina extending to near basal 3/4 of tergal length, lacking discal carinae, tergite 2 (V) with median carina extending to near basal 1/4 of tergal length, tergites 2–4 (V–VII) each with small basolateral foveae. Sternite 2 (IV) with broad basal sulcus, lacking mediobasal foveae, basolateral foveae developed as large cuticular pockets, with two pairs of antebasal nodules, sternites 3–5 (V–VII) with basolateral foveae, one median and two lateral nodules, sternite 7 (IX) nearly oval, with well-sclerotized apical half and less sclerotized basal half.

Aedeagus (Fig. 8H–J) 0.57 mm long, with slightly asymmetric median lobe, apical part of median lobe narrowed, apex broadly truncate in dorso-ventral view; endophallus lacking strongly sclerotized structures, composed of broad, elongate membrane with numerous small denticles.

**Female.** Unknown.

#### Comparative notes.

*Horniella sabahensis* sp. nov. is placed as a member of the *H. hirtella* group. Males of this species share with *H. prolixo* Yin & Li from Thailand the weakly developed anterolateral genal projections, lack of an expansion at the lateral margin of the scape, and a moderately expanded preapical portion of the metatibia. They can be best separated by the larger body size (3.41 mm vs 2.95–3.02 mm), tergite V with a short median carina (lacking in *H. prolixo*), as well as the much narrower apex of the aedeagus of the new species.

#### Distribution.

East Malaysia: Sabah.

#### Etymology.

The new species is named after its type locality, Sabah, East Malaysia.

### Horniella taiwanensis

Taxon classificationAnimaliaAmphipodaHornelliidae

Zhang, Hu & Yin sp. nov.

2C8404EE-EEEF-503F-A23F-EC60DF39E198

http://zoobank.org/903D13DA-D767-4259-83A3-3C02BF9DD0E0

Figures 7B , 9

#### Type material.

***Holotype*: China**: ♂, ‘TAIWAN: Taoyuan City, Northern Cross-island Highway 35.7 k (北横公路35.7 k), Fusing Township, 15-IV-2018, leg. K. X. Zhan’ (NMNS). ***Paratypes*: China**: 1 ♂, ‘TAIWAN: Nantou County, Sun Moon Lake (日月潭), Yuchih Township, 13-XII-2016, leg. F. C. Hsu’ (NMNS); 1 ♂, ‘TAIWAN: Taichung City, Dakeng (大坑), Xinshe Dist., 24.1932, 120.7991, 10-IV-2021, leg. C. T. Hsu (under rock)’ (NMNS); 1 ♂, ‘Kuantaoshan, NANTOU, TAIWAN, 南投县关刀山, 16.vii.1999, M. Tanikado leg.’ (MHNG); 1 ♂, ‘Tehuashe (800 m), NANTOU, TAIWAN, 南投县德化社, 2.vii.2000, H. Y. Chu leg. (at light) (MHNG); 1 ♂, ‘Taiwan, Nantou, Meifeng (梅峰), 2100 m, 6.v.01 (sifting of litter), Sugaya lgt.’ (SNUC).

#### Diagnosis.

**Male.** Head longer than wide, with distinct anterolateral genal projections, anterior margin of projection narrowly emarginate, with long ocular canthus; scape roundly expanded at basolateral margin, antennomeres 9–11 slightly enlarged. Pronotum rounded at anterolateral margins. Protrochanter, profemur and mesotrochanter each with ventral spine; protibia and mesotibia with large apical projection. Tergite 1 (IV) with median carina extending posteriorly for approximately 1/4 of tergal length, lacking discal carinae. Aedeagus with asymmetric median lobe, right half of median lobe greatly protruding apicad, apical margin nearly rounded in dorsal view.

#### Description.

**Figure 9.**
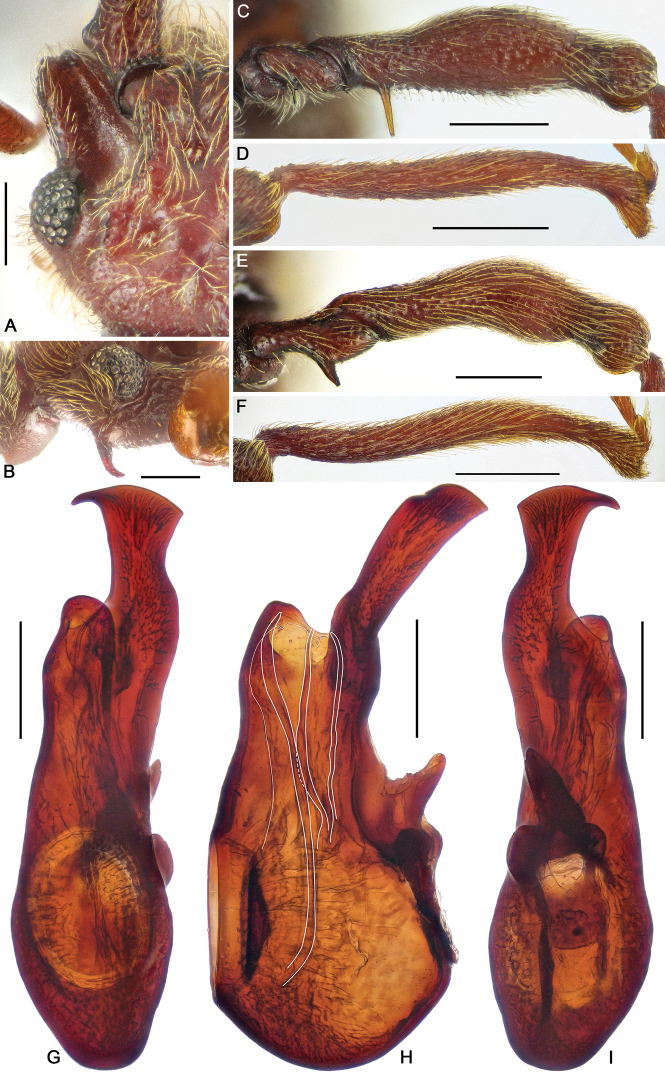
Diagnostic characters of *Horniella taiwanensis* sp. nov. **A** left half of head, in dorsal view **B** head, in lateral view **C** protrochanter and profemur **D** protibia **E** mesotrochanter and mesofemur **F** mesotibia **G–I** aedeagus, in dorsal (**G**), lateral (**H**), and ventral (**I**) view. Scale bars: 0.2 mm (**A, B, G–I**); 0.3 mm (**C–F**).

**Male.** Body reddish-brown, length 4.05–4.15 mm. Head (Fig. 9A) slightly longer than wide, HL 0.84–0.87 mm, HW 0.74–0.76 mm; anterolateral genal projection distinct, anterior margin of projection narrowly emarginate; antenna 2.1 mm long, scape roundly expanded at basolateral margin, antennomeres 2–8 slightly elongate or moniliform, club loosely formed by apical three moderately enlarged antennomeres, antennomere 11 largest, slightly shorter than antennomeres 9 and 10 combined; indistinct lateral postantennal pits present; eyes prominent, each composed of approximately 40 large facets, with long ocular canthus (Fig. 9B).

Pronotum slightly longer than wide, PL 0.78–0.82 mm, PW 0.76–0.77 mm; widest at apical 1/3; anterolateral margins rounded; disc moderately convex, finely punctate, with distinct median antebasal and lateral antebasal foveae connected by complete transverse antebasal sulcus.

Elytra much wider than long, EL 0.94–1.01 mm; EW 1.51 mm; each elytron with two large, setose basal foveae; discal striae extending from outer basal foveae to apical 2/3 of elytral length.

Legs elongate; protrochanter (Fig. 9C) with short, acute ventral spine, profemur (Fig. 9C) with distinctly long ventral spine near base; protibia (Fig. 9D) with large apical projection; mesotrochanter (Fig. 9E) with sharp ventral spine, mesofemur (Fig. 9E) simple, mesotibia (Fig. 9F) with moderately large projection.

Abdomen approximately as long as broad, broadest at lateral margins of tergite 1 (IV), AL 1.45–1.49 mm, AW 1.47–1.49 mm; tergite 1 (IV) slightly longer than tergites 2 (V), with short median carina extending to near basal 1/4 of tergal length, lacking discal carinae, tergite 2 (V) lacking carina, tergites 2–4 (V–VII) each with small basolateral foveae. Sternite 2 (IV) with broad basal sulcus, lacking mediobasal foveae, basolateral foveae developed as large cuticular pockets, with two pairs of antebasal nodules, sternites 3–5 (V–VII) with basolateral foveae, one median and two lateral nodules, sternite 7 (IX) with well-sclerotized apical half and less sclerotized basal half.

Aedeagus (Fig. 9G–I) 1.01 mm long, with strongly asymmetric median lobe, right half of median lobe greatly protruding apicad, apex broadened, with round apical margin dorso-ventral view; endophallus composed of one elongate, and two much shorter sclerites.

**Female.** Unknown.

#### Comparative notes.

This species is placed as a member of the *H. centralis* group and is most similar to *H. sichuanica* Yin & Li in the shapes of the anterolateral genal projections and spination of the legs. They can be clearly separated by the larger body size (4.05–4.15 mm vs 3.58–3.77 mm), the more distinct apical projections of protibia and mesotibia, and the dilated apex of the aedeagal median lobe of the new species.

#### Distribution.

China: Taiwan.

#### Etymology.

The new specific is named after Taiwan.

**Figure 10.**
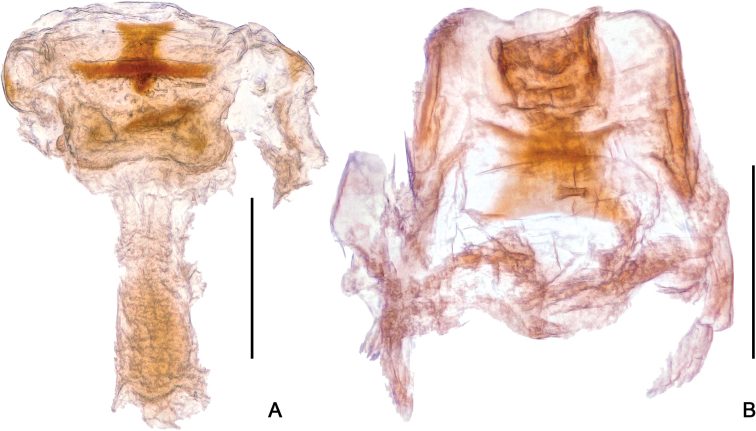
Female genitalia of *Horniella* species, in dorsal view **A***H. bifurca* sp. nov. **B***H. nantouensis* sp. nov. Scale bars: 0.2 mm.

##### List of *Horniella* species worldwide

*H. aculeata* Yin & Li, 2015: 110. China: Yunnan; Thailand: Nan, Mae Hong Son.

*H. asymmetrica* Yin & Li, 2014: 42. Thailand: Prachin Buri, Chanthaburi.

*H. awana* Yin & Li, 2014: 65. West Malaysia: Pahang.

*H. bifurca* Zhang, & Yin, sp. nov. Thailand: Chiang Mai.

*H. burckhardti* Yin & Li, 2014: 45. Thailand: Chiang Mai.

*H. centralis* Yin & Li, 2014: 11. China: Shaanxi.

*H. cibodas* Yin & Li, 2014: 74. Indonesia: West Java.

*H. confragosa* Yin & Li, 2014: 14. China: Guangxi, Guizhou.

*H. dao* Yin & Li, 2014: 17. China: Sichuan.

*H. falcis* Yin & Li, 2014: 18. China: Guizhou.

*H. gigas* Yin & Li, 2014: 66. East Malaysia: Sabah.

*H. haucki* Zhang, & Yin, sp. nov. Thailand: Mae Hong Son.

*H. himalayica* Yin & Li, 2014: 35. Nepal: Bāgmatī añcal; India: Uttarakhand.

*H. hirtella* (Raffray, 1901: 30). Sri Lanka: Northern, North Central, Central, Uva.

*H. hongkongensis* Yin & Li, 2014: 21. China: Hong Kong.

*H. intricata* Yin & Li, 2014: 47. Thailand: Mae Hong Son, Chiang Mai.

*H. jinggangshana* Yin & Li, 2015: 113. China: Jiangxi.

*H. kaengkrachan* Yin & Li, 2014: 50. Thailand: Phetchaburi.

*H. khaosabap* Yin & Li, 2014: 51. Thailand: Chanthaburi.

*H. khasiensis* Zhang, & Yin, sp. nov. India: Meghalaya.

*H. loebli* Yin & Li, 2014: 54. Thailand: Chiang Mai.

*H. nakhi* Yin & Li, 2014: 25. China: Yunnan.

*H. nantouensis* Zhang, Hu & Yin, sp. nov. China: Taiwan.

*H. philippina* Yin & Li, 2014: 63. Philippines: Laguna.

*H. phuphaman* Yin & Li, 2014: 56. Thailand: Khon Kaen.

*H. pilosa* Yin & Li, 2014: 69. East Malaysia: Sabah.

*H. prolixo* Yin & Li, 2014: 60. Thailand: Chiang Mai.

*H. sabahensis* Zhang, & Yin, sp. nov. East Malaysia: Sabah.

*H. schuelkei* Yin & Li, 2014: 25. China: Yunnan.

*H. schwendingeri* Yin & Li, 2014: 60. Thailand: Chiang Mai.

*H. sichuanica* Yin & Li, 2014: 28. China: Sichuan.

*H. simplaria* Yin & Li, 2014: 28. China: Guangxi.

*H. smetanai* Yin & Li, 2014: 72. East Malaysia: Sabah.

*H. taiwanensis* Zhang, Hu & Yin, sp. nov. China: Taiwan.

*H. tianmuensis* Yin & Li, 2014: 32. China: Zhejiang.

## Supplementary Material

XML Treatment for Horniella aculeata

XML Treatment for Horniella bifurca

XML Treatment for Horniella haucki

XML Treatment for Horniella khasiensis

XML Treatment for Horniella nantouensis

XML Treatment for Horniella sabahensis

XML Treatment for Horniella taiwanensis
